# Characterization of *Aspergillus nidulans* Biofilm Formation and Structure and Their Inhibition by Pea Defensin *Ps*d2

**DOI:** 10.3389/fmolb.2022.795255

**Published:** 2022-01-27

**Authors:** Caroline Corrêa-Almeida, Luana P. Borba-Santos, Rodrigo Rollin-Pinheiro, Eliana Barreto-Bergter, Sonia Rozental, Eleonora Kurtenbach

**Affiliations:** ^1^ Laboratório de Biologia Molecular e Bioquímica de Proteínas, Programa de Biologia Molecular e Estrutural, Instituto de Biofísica Carlos Chagas Filho, Universidade Federal Do Rio de Janeiro, Rio de Janeiro, Brasil; ^2^ Laboratório de Biologia Celular de Fungos, Programa de Parasitologia e Biologia Celular, Instituto de Biofísica Carlos Chagas Filho, Universidade Federal Do Rio de Janeiro, Rio de Janeiro, Brasil; ^3^ Laboratório de Química Biológica de Microrganismos, Departamento de Microbiologia Geral, Instituto de Microbiologia Paulo de Góes, Universidade Federal Do Rio de Janeiro, Rio de Janeiro, Brasil

**Keywords:** fungal biofilms, biofilm structure, Aspergillus nidulans, Pisum sativum defensin 2, plant defensins, antimicrobial peptides (AMPs), antibiofilm activity, antibiofilm therapy

## Abstract

Approximately four million people contract fungal infections every year in Brazil, primarily caused by *Aspergillus spp*. The ability of these fungi to form biofilms in tissues and medical devices complicates treatment and contributes to high rates of morbidity and mortality in immunocompromised patients. *Ps*d2 is a pea defensin of 5.4 kDa that possesses good antifungal activity against planktonic cells of representative pathogenic fungi. Its function depends on interactions with membrane and cell wall lipid components such as glucosylceramide and ergosterol. In the present study, we characterized *Aspergillus nidulans* biofilm formation and determined the effect of *Ps*d2 on *A. nidulans* biofilms. After 4 hours, *A. nidulans* conidia adhered to polystyrene surfaces and formed a robust extracellular matrix-producing biofilm at 24 h, increasing thickness until 48 h *Ps*d2 inhibited *A. nidulans* biofilm formation in a dose-dependent manner. Most notably, at 10 μM *Ps*d2 inhibited 50% of biofilm viability and biomass and 40% of extracellular matrix production. *Ps*d2 significantly decreased the colonized surface area by the biofilm and changed its level of organization, causing a shortening of length and diameter of hyphae and inhibition of conidiophore formation. This activity against *A. nidulans* biofilm suggests a potential use of *Ps*d2 as a prototype to design new antifungal agents to prevent biofilm formation by *A. nidulans* and related species.

## 1 Introduction

Plant defensins are an extensive family of cationic and cysteine-rich peptides of 45–54 amino acid residues that participate in the defense mechanism of various organisms against a wide range of microorganisms (Lay and Anderson, 2005; Vriens et al., 2014; Cools et al., 2017). Other activities, such as anti-tumoral, have also been described (Hoskin and Ramamoorthy, 2008; Deslouches and Di, 2017; Do Amaral et al., 2020; Tornesello et al., 2020). Defensins are composed of three antiparallel β-sheets and one α-helix stabilized by four or five disulfide bonds (Almeida et al., 2000; Lacerda et al., 2014; Vriens et al., 2014). The first and crucial step in plant defensin activity is an interaction with membrane targets such as sphingolipids and phospholipids (Cools et al., 2017; Parisi et al., 2019). Fungal glucosylceramide-enriched membranes are critical targets for some plant defensins. The presence of glucosylceramide in *Candida albicans* is essential for producing reactive oxygen species, which damages DNA, RNA, and proteins, leading to apoptosis after challenge with the radish defensin *Rs*AFP2 (Aerts et al., 2007; Aerts et al., 2009; Thevissen et al., 2012). *Ps*d1 is a defensin isolated from seeds of the garden peas (*Pisum sativum*) (Almeida et al., 2000) and interacts with glucosylceramide and ergosterol from *Aspergillus spp.* (De Medeiros et al., 2014; Fernandes et al., 2016)*.* The defensin is internalized and transported to the nucleus, where it interacts with cyclin F, a protein involved in controlling the cell cycle (Lobo et al., 2007; De Medeiros et al., 2014; Fernandes et al., 2016).

*Ps*d2 (6NOM PDB ID) is a 47-amino-acid peptide isolated from pea seeds. This molecule possesses inhibitory activity against several planktonic fungal species, including *Aspergillus nidulans*, *Aspergillus niger*, *Neurospora crassa*, and *Fusarium solani* (Almeida et al., 2000; Amaral et al., 2019). Like other defensins, the structure of *Ps*d2 includes α-helix and antiparallel β-sheets stabilized for four disulfide bonds formed from eight cysteine residues (Pinheiro-Aguiar et al., 2019). Recently, our group showed that *Ps*d2 interacts preferentially with mimetic membranes composed of the fungal specific lipids glucosylceramide and ergosterol as opposed to membranes containing lipids present on mammalian cells (Amaral et al., 2019). *Ps*d2 activity also depends on the presence of glucosylceramide because mutants of *A. nidulans* and *C. albicans* whose glucosylceramide synthase gene (Δgcs) are deleted were less susceptible to *Ps*d2 than wild-type strains (Amaral et al., 2019).

The antifungal activity of plant defensins has been studied for several decades; nevertheless, their antibiofilm activity, including activity mediated *Ps*d2, has not been explored. Biofilm formation by filamentous fungi begins with the conidial attachment on biotic or abiotic surfaces, followed by germination and surface colonization. Mature biofilms show a complex architecture characterized by water channels and extracellular matrix (ECM) (Harding et al., 2009; González-Ramírez et al., 2016; Gulati and Nobile, 2016; Wall et al., 2019). Generally, biofilms create an impermeable barrier that decreases drug delivery, contributing to the therapeutic failure (Nett et al., 2007; Harding et al., 2009; Mitchell et al., 2016a; Perlin et al., 2017).

One of the most studied fungal infections associated with biofilm formation is aspergillosis, a set of allergic and invasive syndromes caused by *A. fumigatus*, *A. flavus*, *A. terreus*, *A. niger,* and *A. nidulans* (Richardson and Medicine, 2008; McCarthy and Walsh, 2017). *Aspergillus* conidia are present in the air, soil, food products, indoor environments, and several varieties of organic debris that can be easily aerosolized and inhaled into the human respiratory tract. Thus, *Aspergillus spp.,* after penetrating the pulmonary epithelium, show a complex and multicellular structure and cause systemic infections, primarily in immunocompromised patients (Richardson and Medicine, 2008; Ramage et al., 2011). *Aspergillus* spp. can either enter the host through alternative routes resulting in severe biomaterial-related infections in catheters, joint replacements, cardiac pacemakers, heart valves replacement, and breast implants, all of which provide ideal niches for biofilm formation (Ramage et al., 2011; Nett and Andes, 2015).

In immunocompromised patients, *A. nidulans* is an opportunistic fungal pathogen; it has been widely used as a model organism to study developmental processes (Ojeda-López et al., 2018; Bastos et al., 2020). *A. nidulans* is phylogenetically related to other species of economic importance, including *A. niger* and *A. oryzae* and clinically relevant species such as *A. fumigatus* (Galagan et al., 2005)*.* Previous studies have described the *A. nidulans* adhesion in hydrophobic surfaces and their biofilm development, both of which are critical for the virulence of *Aspergillus* species (Shukla et al., 2017; He et al., 2018; Kadry et al., 2018; Lingo et al., 2021). Kadry et al. (2018) used scanning electron microscopy (SEM) and found that *A. nidulans* strains (∆*gmt*A and ∆*gmt*B) did not produce guanosine diphosphate mannose transporter (GMT), a transporter required for mannosylation of galactomannan and mannoproteins, exhibited smaller colony size, reduced sporulations, increased hyphal width and decreased based cell length. The disturbances could explain these defective phenotypes during cell wall biosynthesis. In addition, the spores of *A. nidulans* ∆*gmt*A had a rougher surface than the wild-type strain, being less adhesive to polystyrene plates, reducing the biofilm formation (Kadry et al., 2018). He et al. (2018) evaluated the role of α-1,3-glucan, a primary cell wall component in many filamentous fungi, including in *A. nidulans* biofilm formation. The overexpression of α-1,3-glucan synthase in *A. nidulans* triggered a higher cellular adhesion to hydrophobic materials and biofilm formation than the wild-type strain, which could contribute to fungal virulence (He et al., 2018). Those findings may indicate that cell adhesion to hydrophobic surfaces can contribute, and it is required to the biofilm formation of *A. nidulans* (He et al., 2018; Kadry et al., 2018).

Differently, in the present study, we described the stages (adhesion, filamentation, maturation, and ECM production) of *A. nidulans* biofilm formation and assessed the potential antibiofilm activity of the defensin *P*sd2. We have used phase-contrast optical microscopy, spinning disk confocal microscopy (SDCM), and scanning electron microscopy (SEM) techniques to characterize the *A. nidulans* biofilm formation. The results showed that *A. nidulans* conidia adhered to polystyrene plates at 4 h, germinated, and colonized the surface in up to 20 h. The SDCM revealed that *A. nidulans* formed a robust extracellular matrix-producing biofilm at 24 h, increasing in thickness until 48 h. Optical density analysis, XTT-reduction, crystal violet, and safranin O assays detected that *Ps*d2 defensin inhibited *A. nidulans* biofilm formation in a dose-dependent manner, most notably at 10 μM, which inhibited 50% of biofilm viability and biomass and 40% of extracellular matrix production. *Ps*d2 significantly reduced the colonized polystyrene and central venous catheter (CVC) areas by the biofilm and changed its level of organization, causing a shortening of length and diameter of hyphae and inhibition of conidiophore formation.

## 2 Materials and Methods

### 2.1 *Ps*d2 Recombinant Expression and Purification

Recombinant *Ps*d2 was expressed and purified as described previously (Almeida et al., 2000; Cabral et al., 2003; Amaral et al., 2019). Briefly, an isolated colony of *Pichia pastoris* GS115 containing pPIC9-*Ps*d2 construct was grown in 5 ml of buffered minimal glycerol medium (100 mM potassium phosphate buffer pH 6, 1.34% Difco yeast nitrogen base without amino acids, 4 × 10^−5^% biotin, and 1% glycerol) at 28°C, for 16 h and, subsequently, in 600 ml of buffered minimal glycerol for approximately 18 h. The culture was centrifuged at 5,300 *g* for 10 min and transferred to buffered basal salt medium (100 mM potassium phosphate buffer pH 6, 4 g/L ammonium chloride, 4 × 10^−5^% biotin, 0.68 mM calcium chloride, 1.7 mM sodium chloride, 0.1% magnesium solution (0.7 M magnesium chloride and 2 M magnesium sulfate) and 0.01% trace elements solution (0.24 M citric acid, 0.17 M zinc sulfate, 26 mM iron (II) sulfate, 0.9 mM copper (II) sulfate, 0.3 M magnesium sulfate, 0.81 mM boric acid, 0.21 mM sodium molybdate, 1% chloroform) supplemented with 0.7% methanol and grown for 120 h at 28°C under constant agitation. Recombinant *Ps*d2 expression was induced by the daily addition of 0.7% methanol to the growth culture. The culture was centrifuged, and the supernatant was applied to a HiPrep 26/60 Sephacryl S-100 HR column (GE Healthcare Life Sciences, Little Chalfont, United Kingdom) with a constant flow of 25 mM Tris-HCl pH 7.5 buffer at 1 ml/min. Then, the fractions containing low-molecular-weight peptides were grouped, dried, and applied to a Luna C8 Reverse Phase Column (Phenomenex, Torrance, CA, United States). The elution containing the recombinant *Ps*d2 was conducted using a gradient of linear concentration 20–50% solution B (90% acetonitrile, 0.1% trifluoroacetic acid) at a flow at 4 ml/min. The peptide fractions were dried, and 20 ml of ultrapure water was added, followed by three cycles of freezing and drying. The peptide concentration was estimated using Lowry’s method (Lowry et al., 1951). Finally, peptide purity and molecular mass were confirmed using mass spectrometry at Centro de Espectrometria de Massas de Biomoléculas (CEMBIO-UFRJ).

### 2.2 Fungal Strain and Growth Conditions

All experiments were performed using *A. nidulans* GR5 (Fernandes et al., 2016). *A. nidulans* GR5 was maintained in YUU agar (0.5% yeast extract, 2% glucose, 1.2% uridine, 1.2% uracil, 0.1% trace elements solution containing 75 mM zinc sulfate heptahydrate, 180 mM boric acid, 25 mM manganese (II) chloride tetrahydrate, 6 mM cobalt (II) chloride heptahydrate, 6 mM copper (II) sulfate pentahydrate, 1 mM ammonium molybdate tetrahydrate, 140 mM ethylenediaminetetraacetic acid and 1.7% agar) in Petri dishes at 37°C for 72 h. Conidia were scraped off and diluted in phosphate-buffered saline (PBS) buffer (137 mM sodium chloride, 2.7 mM potassium chloride, 10 mM dibasic sodium phosphate, 1.8 mM potassium phosphate monobasic, pH 7.0) containing 0.1% Tween 80. This suspension was filtered with sterile gauze pads, vortexed vigorously for 1 min, and centrifuged at 3,470 *g* at 4°C for 5 min. The supernatants were discarded, and a new volume of PBS was added. This step was repeated twice. Then, the conidial suspension was standardized to 1 × 10^4^ conidia/mL in different media, depending on the experiment.

### 2.3 Biofilm Formation and Quantification

*A. nidulans* biofilms were cultivated in different media from the conidial suspension standardized to 1 × 10^4^ conidia/mL. Three culture media were tested: RPMI medium (Sigma-Aldrich, St. Louis, United States) buffered with morpholinepropanesulfonic acid to pH 7.0, RPMI UU medium pH 7.0 (RPMI medium supplemented with 1.2% uridine and 1.2% uracil), and YUU medium pH 5.9. Biofilms were formed in polystyrene 96-well plates (NEST Biotechnology Co., Wuxi, China) and incubated at 37°C for 12, 24, 36, 48, 60, and 72 h. At each time, the biofilms were carefully washed with PBS pH 7.0 and quantified using two methods: XTT (2,3-bis-(2-methoxy-4-nitro-5-sulphonyl)-2H-tetrazolium-5-carboxanilide) (ThermoFisher Scientific, Waltham, MA, United States) to measure the metabolic activity and crystal violet (ThermoFisher Scientific) to measure the global cell mass. Four independent experiments were performed in triplicate.

### 2.4 Antibiofilm Activity of *Ps*d2

The effect of *Ps*d2 in *A. nidulans* biofilm formation was evaluated after exposure for 48 h *Ps*d2 (1.25–40 µM), PBS pH 7.0 or 10 μM itraconazole was added to polystyrene 96-well plates (NEST Biotechnology Co., Wuxi, China) containing 1 × 10^4^ conidia/mL diluted in YUU medium pH 5.9. Biofilm was incubated for 48 h at 37°C, washed with PBS pH 7.0, and quantified using XTT-reduction assay, crystal violet, or safranin O (Sigma-Aldrich, St. Louis, United States) to measure and follow the presence of ECM. The percentage of growth inhibition was calculated considering the culture absorbance in the presence of PBS pH 7.0 or the presence of 10 μM itraconazole as 0 and 100% inhibition, respectively. Four independent experiments were performed in triplicate.

### 2.5 XTT-Reduction Assay

The biofilms were quantified by XTT-reduction as previously described (Pierce et al., 2008) with modifications. Biofilms described in items 2.3 and 2.4 were washed with PBS pH 7.0 once, and then a solution containing 0.5 mg/ml XTT and 6.4 μg/ml menadione was added. The microplate was incubated at 37°C for 2 h, protected from the light. The final solution was transferred to a new microplate, and the absorbance was measured at 490 nm using an Asys UVM340 microplate reader (Biochrom, Cambridge, United Kingdom).

### 2.6 Crystal Violet Assay

The biofilms were quantified by crystal violet as described (Silva et al., 2009). The biofilms were washed with PBS pH 7.0 once, and then a solution containing 0.02% crystal violet was added. The microplates were incubated at room temperature for 5 min. The solution was discarded, and the wells were washed twice with PBS, pH 7.0. Ethyl alcohol was added to solubilize the crystal violet. The colored solution was transferred to a new microplate. The absorbance was measured at 565 nm using an Asys UVM340 microplate reader (Biochrom Cambridge, United Kingdom).

### 2.7 Safranin O Assay

The biofilms were quantified by safranin O as described (Mello et al., 2018) with modifications. A solution containing 1% safranin O was added to the biofilms. The microplate was incubated at room temperature for 5 min. The colored solution was discarded, and the biofilms were washed five times with PBS, pH 7.0. The colored solution was transferred to a new microplate at the final washing. The absorbance was measured at 492 nm using an Asys UVM340 microplate reader (Biochrom, Cambridge, United Kingdom).

### 2.8 Phase-Contrast Microscopy and Optical Density Analysis

*A. nidulans* biofilms were grown in polystyrene 24-well plates (TPP, Trasadingen, Switzerland) from 10^4^ conidia/mL cultivated in RPMI, RPMI UU, and YUU media in the presence of PBS pH 7.0. Biofilms were also grown in the presence of 5, 10, and 20 µM *Ps*d2 diluted in the YUU medium. The plates were sealed using optical adhesive film (Life Technologies, CA, United States) and incubated at 37°C in the Cytation 5 Cell Imaging Multi-Mode Reader (BioTek, VT, United States). Growth kinetics were analyzed using images from the phase-contrast microscopy (at 20 x magnification) and by optical density (at 540 nm). Images were captured every hour in the same field for 24 h, while optical density values were obtained every hour for 72 h. Two independent experiments were performed in triplicate.

### 2.9 SDCM

*A. nidulans* biofilms were grown from 1 × 10^4^ conidia/mL cultivated in cell-view glass-bottom culture dishes with four compartments (Cell View, Greiner Bio-One, Germany) for 24, 39, and 48 h in the presence of PBS pH 7.0 in YUU medium. Biofilms were also grown in the presence of 10 µM *Ps*d2 for 24 h in YUU medium. At designated times, the biofilms were washed once with PBS (pH 7.0) and incubated with Filmtracer Sypro Ruby (Invitrogen, California, United States) for 30 min at 37°C and 10 μg/ml Calcofluor white (Sigma-Aldrich St. Louis, United States) for 15 min at room temperature. Filmtracer Sypro Ruby binds to glycoproteins from ECM, while Calcofluor white binds to chitin structures in the cell wall of the fungi. The biofilms were observed at 20x magnification using a Zeiss Cell Observer Yokogawa spinning disk confocal microscope (Cell Observed SD, Carl-Zeiss, Oberkochen, Germany). Two experiments were performed in duplicate.

### 2.10 SEM

For SEM observation, *A. nidulans* biofilms were grown in central venous catheters (CVCs) (BD Intracath™ Vialon™, Franklin Lakes, United States) with sections of 0.5 cm. The CVCs were placed into polystyrene 96-well plates, and 1 × 10^4^ conidia/mL in YUU medium were added. These sets were incubated for 24 h in the presence of 10 µM *Ps*d2 or PBS pH 7.0 for untreated cells. The biofilms adhered to the CVCs were washed with PBS and fixed in 2.5% glutaraldehyde and 4% formaldehyde in 0.1 M cacodylate buffer for 1 h at room temperature. Subsequently, the biofilms were post-fixed in 1% osmium tetroxide and 1.25% potassium ferrocyanide for 30 min and then dehydrated in increasing ethanol concentrations (30, 50, 70, 90, and 100%) for 15 min at each concentration. The samples were critical-point-dried in CO_2_ (EM CPD300, Leica, German), coated with gold, and observed using an FEI Quanta 250 scanning electron microscope (FEI, Netherlands). The diameters of 100 hyphae in five different fields of untreated and treated biofilms were analyzed and determined using ImageJ Software.

### 2.11 Statistical Analysis

All statistical analyses were performed using GraphPad Prism 8.2 (GraphPad Software, San Diego, CA, United States). Two-way ANOVA and Tukey’s multiple comparisons tests were applied to analyze the growth kinetics of *A. nidulans* in RPMI UU and YUU media. An unpaired *t*-test was applied to analyze the hyphae diameter in the untreated and *Ps*d2 treated biofilms. One-way ANOVA and Tukey’s multiple comparisons tests were applied to analyze the thickness average of the biofilms grown in 24, 39, and 48 h. Differences were significant when the *p*-value was less than 0.05.

## 3 Results

### 3.1 YUU Is the Best Medium to Grow A. nidulans Biofilms

**FIGURE 1:**
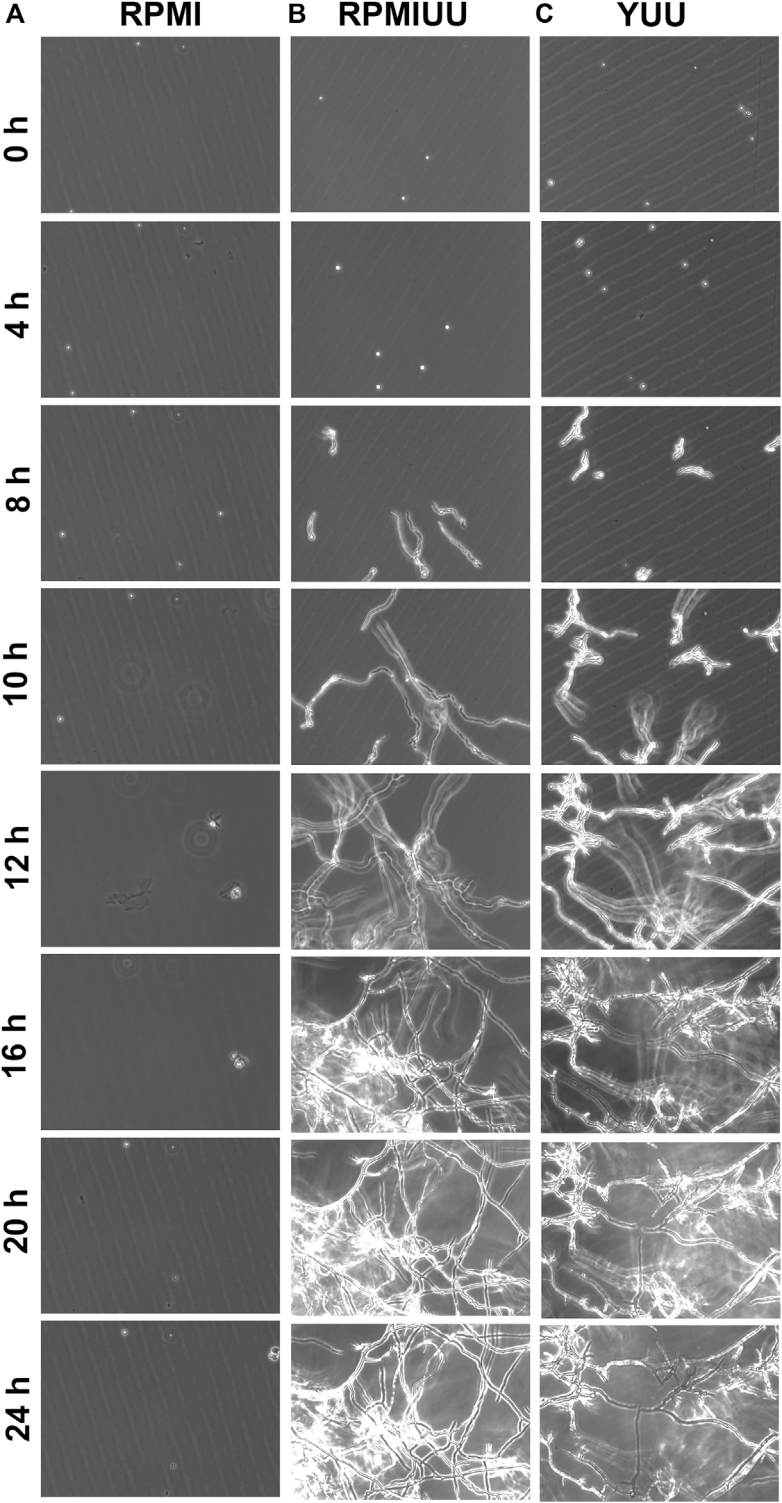
Phase-contrast micrographs of biofilm formation in RPMI, RPMI UU, and YUU media. 1 × 10^4^ conidia of *A. nidulans* were grown in RPMI **(A)**, RPMI UU **(B),** and YUU **(C)** media in polystyrene 24-well plates for 24 h at 37°C. The images were captured every hour from the same field using Cytation-5 (Biotek). Magnification of 20 x was used in all panels.

First, three media (RPMI, RPMI UU, and YUU) were tested to observe the growth and formation of *A. nidulans* biofilms on polystyrene substrates. To analyze the initial phases of *A. nidulans* biofilm formation, the fungus was cultivated in RPMI, RPMI UU, and YUU media for 24 h, and the optical density was accompanied using Cytation 5 equipment (Figure 1). *A. nidulans* conidia incubated in RPMI medium did not adhere to the polystyrene surface and did not germinate (Figure 1A), whereas a robust biofilm was observed in RPMI UU and YUU media (Figures 1B,C). In this latter condition, the conidia adhered at 4 h and germinated into hyphae, from 4 to 8 h. Mycelia development and expansion were observed between 10 and 12 h, with hyphae-hyphae adhesion and interconnections in 16–24 h, generating a very dense network. After 48 h, due to the high density and thickness, the biofilm has detached to the polystyrene surface (data not shown).

**FIGURE 2:**
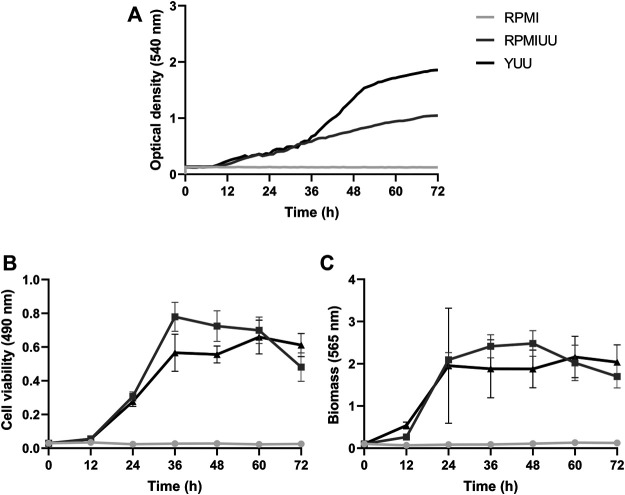
*A. nidulans* biofilm formation in RPMI, RPMI UU, and YUU media. 10^4^ conidia/mL were incubated in RPMI, RPMI UU, and YUU media in polystyrene 96-well plates for 72 h at 37°C. **(A)** Optical density analysis; **(B)** Cell viability analysis; and **(C)** Biomass quantification of *A. nidulans* biofilm formation over 72 h. Error bars represent standard deviations from the mean of two independent experiments performed in triplicate. Significant differences (*p* < 0.05) are represented by asterisks when the RPMI UU growth kinetics were compared to the YUU medium. **p < 0.01; ****p < 0.0001.

The growth kinetics evaluation was extended for 72 h, and quantifications were performed according to the optical density using a Cytation 5 Cell Imaging Multi-Mode Reader (Figure 2A), the XTT-reduction assay (Figure 2B), and the crystal violet assay (Figure 2C). According to these three methodologies, *A. nidulans* did not form biofilms in the RPMI medium until 72 h (Figures 1A–C). Considering cell density results (Figure 2A), a denser biofilm was observed in the YUU medium, in which it was possible to observe the classic phases of fungal growth: lag (0–35 h), exponential (36–50 h), and stationary (51–72 h) phases with a mean time of 39 h. However, it was impossible to observe the same response in the RPMI UU medium because of the slower development of the *A. nidulans* biofilm over 72 h.

According to metabolic activity assays (Figure 2B)*, A. nidulans* biofilm growth in RPMI UU medium reached the stationary phase approximately at 36 h when it started to significantly decrease the cell viability until 72 h. *A. nidulans* developed a more viable biofilm in the YUU medium, reaching the stationary phase at 60 h, which started to show a very small decrease in the experimental time.

The total biomass (cell plus ECM) (Figure 2C) of *A. nidulans* biofilm was significantly higher in the RPMI UU than YUU from 36 to 48 h, with a slight decrease until 72 h in RPMI UU media. The biofilm had a similar biomass level from 24 to 72 h when grown in the YUU medium, with no significant differences. These data suggest that YUU was the best-tested medium to form *A. nidulans* biofilm due to its composition and the higher cell viability and biomass stability over time. For this reason, the YUU medium was chosen for the biofilm inhibition assays in the presence of *Ps*d2.

### 3.2 A. nidulans Form a Dense Biofilm and Produce an ECM in the YUU Medium

**FIGURE 3:**
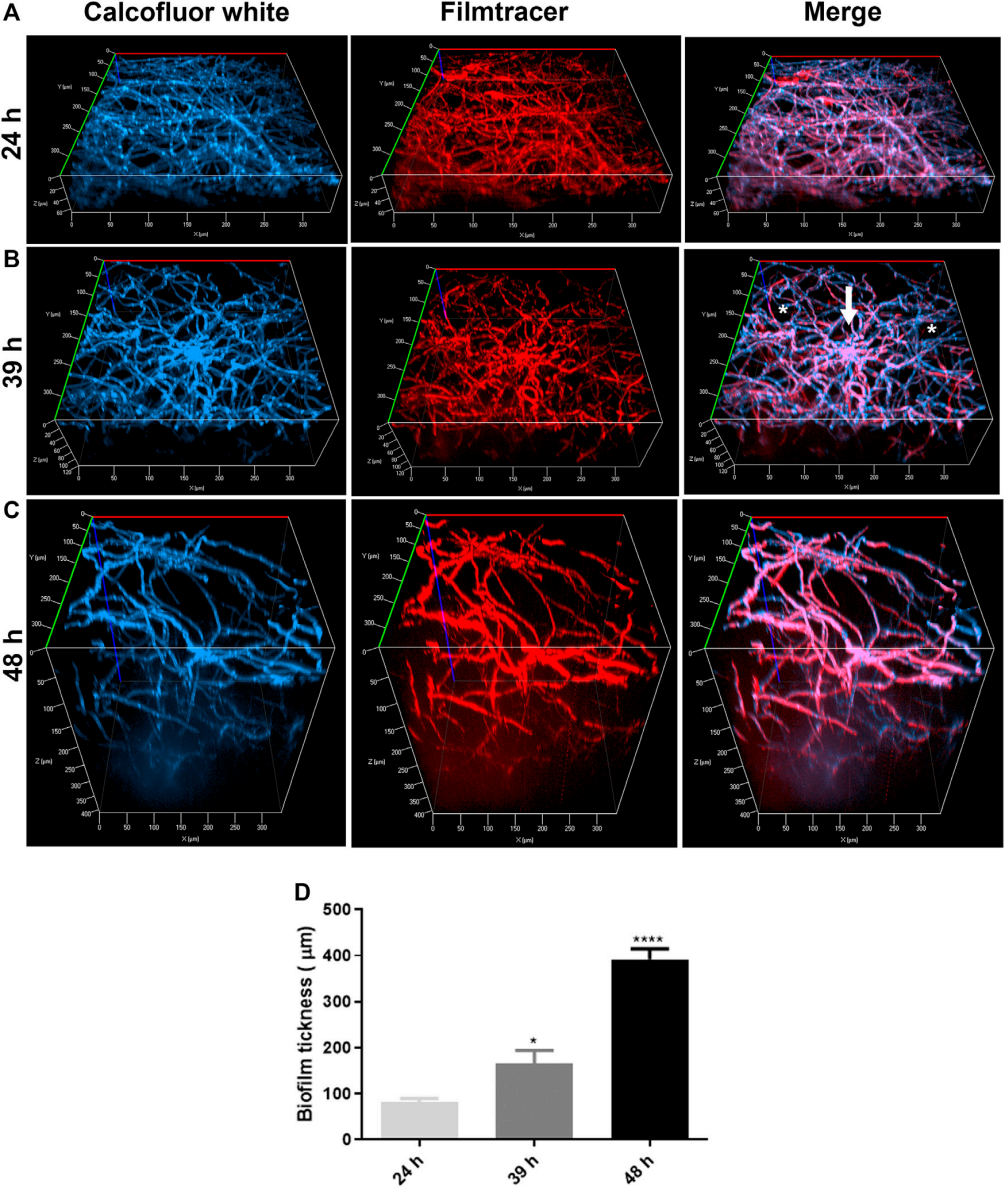
Confocal fluorescence microscopy images of *A. nidulans* biofilms formed in 24, 39, and 48 h. 1 × 10^4^ conidia/mL were incubated in YUU medium in cell-view glass-bottom culture dishes at 37°C for **(A)** 24, **(B)** 39, and **(C)** 48 h. After each time, cell walls were stained with Calcofluor white (first column), and the glycoproteins from the extracellular matrix were stained with Filmtracer Sypro Ruby (second column). The third column represents the merge view of Calcofluor white and Filmtracer Sypro Ruby signals. The images showed the three-dimensional reconstruction of the biofilm formed at each time. The asterisks represent the water channels, whereas the arrow represents the central structure of the biofilm. Magnification of 20x was used in all panels. **(D)** Thickness average of *A. nidulans* biofilm at 24, 39, and 48 h. The average was calculated by measuring the length of the *z*-axis (µm) of three fields for each time of biofilm formation. Error bars represent standard deviations from the mean of two independent experiments performed in duplicate. Statistical differences (*p* < 0.05) are represented by asterisks, for comparisons between 39-h biofilms and 24-h biofilms and between 48-h biofilms and 39-h biofilms; *p < 0.05, ****p < 0.0001.

Confocal fluorescence microscopy was performed to evaluate the biofilm morphology and the ECM production in initial and late times. In glass-bottom dishes, *A. nidulans* biofilms were grown in YUU medium at 37°C for 24, 39, and 48 h. At each time point, the biofilms were stained with Calcofluor white, followed by Filmtracer Sypro Ruby, and observed using a spinning disk confocal fluorescence microscopy (Figure 3).

It was possible to detect the presence of a dense network of interconnected hyphae at 24 h, occupying the entire surface area of the glass bottom with the presence of an ECM (Figure 3A). At 39 h, the biofilm was composed of more complex structures, presenting longer and wider hyphae, with a higher number of interconnected branches (anastomosis) growing from a central structure, represented by the white arrow. Additionally, it was possible to observe water channels, represented by the asterisks, among the branches (Figure 3B). A more robust biofilm was formed in 48 h, formed with thicker and longer hyphae arranged in a more significant number of layers and, consequently, a larger volume than observed in 24 and 39 h (Figure 3C). The thickness average of the biofilms (*z*-axis) increased proportionally and significantly along the time, with approximately 80 nm in 24 h, 120 nm in 39 h, and 400 nm in 48 h (Figure 3D).

### 3.3 *Ps*d2 Inhibits A. nidulans Biofilm Formation

**FIGURE 4:**
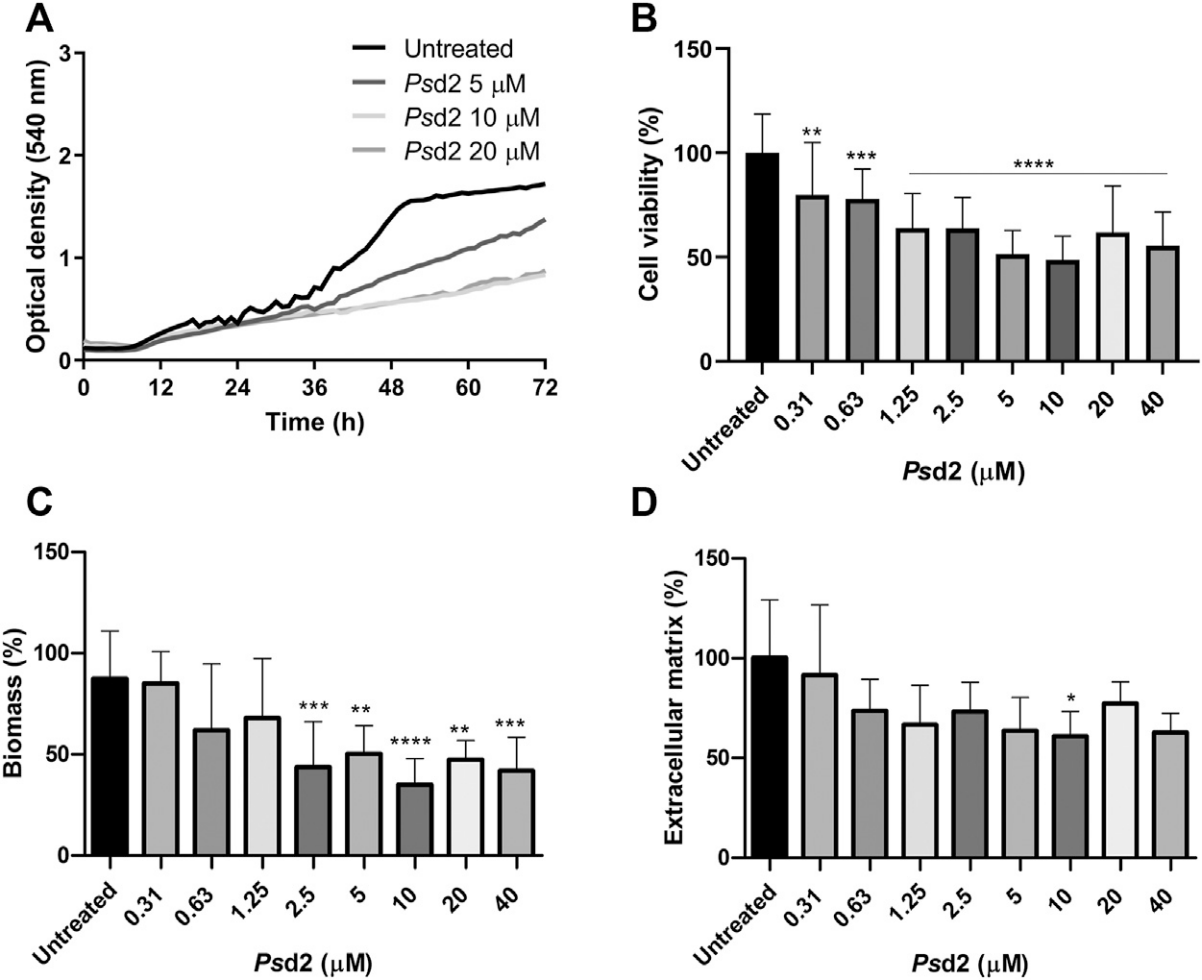
Effect of *Ps*d2 defensin in *A. nidulans* biofilm formation. **(A)** Kinetics of biofilm formation measured by optical density over 72 h. **(B)** Quantification of cell viability by XTT-reduction assay. **(C)** Quantification of global fungal biomass by crystal violet assay. **(D)** Quantification of the extracellular matrix produced with safranin O. The error bars represent standard deviations of the mean of four independent experiments performed in triplicate. Significant differences (*p* < 0.05) are represented by asterisks when the *Ps*d2 concentrations were compared to the untreated control; *p < 0.05; **p < 0.01; ***p < 0.001; ****p < 0.0001.

The effect of *Ps*d2 in biofilm formation was firstly evaluated by optical density during 72 h of treatment with 5, 10, and 20 µM of *Ps*d2 (Figure 4A). Inhibition of biofilm was observed from 36 h, especially when cells were exposed to 10 and 20 μM of *Ps*d2 (Figure 4A). We also measured cell viability, total biomass, and ECM amount after 48 h of treatment with several concentrations of *Ps*d2 (0.31–40 µM). All tested concentrations of *Ps*d2 significantly reduced cell viability of biofilms compared to the control in the absence of the peptide. *A. nidulans* biomass was significantly decreased from *Ps*d2 concentrations higher than 2.5 μM (Figure 4B,C). ECM production was reduced only in the presence of 10 μM of *Ps*d2 (Figure 4D).

### 3.4 *Ps*d2 Reduces the Surface Colonization of A. nidulans Biofilm

**FIGURE 5:**
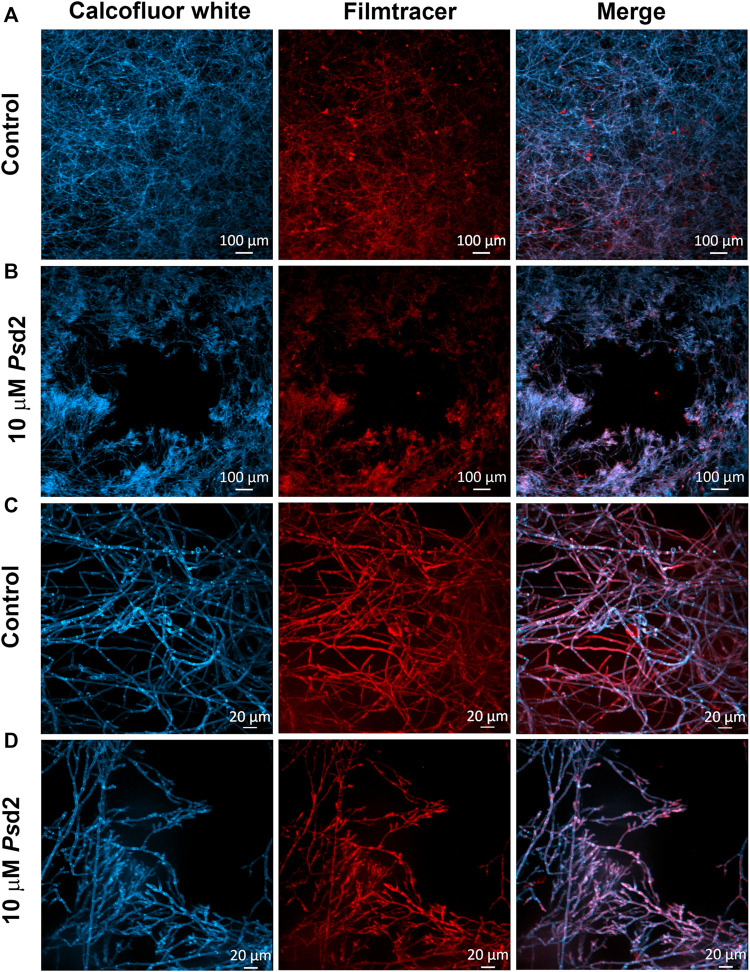
Confocal fluorescence microscopy images of treated *A. nidulans* biofilm with *Ps*d2 defensin. *A. nidulans* biofilms were grown in YUU medium at 37°C for 24 h in glass-bottom dishes in the absence **(A,C)** or presence of 10 µM *Ps*d2 **(B,D)**. After this time point, cell walls were stained with Calcofluor White (first column) and glycoproteins with Filmtracer SyproRuby (second column). The third column represents the merge view by Calcofluor white and Filmtracer Sypro Ruby signals. Magnifications of 5 x **(A,B)** and 20 x **(C,D)** were used in the panels. Scale bars represent 100 µm **(A,B)** and 20 µm **(C,D)**.

Figure 5 shows *A. nidulans* biofilms treated with 10 µM *Ps*d2 for 24 h and analyzed by confocal fluorescence (Figure 5). Staining with Calcofluor white and Filmtracer Sypro Ruby revealed that treated biofilm exhibited smaller surface colonization and gaps showing empty spaces among the biofilm structures adhered on glass-bottom dishes (Figure 5B). Additionally, *Ps*d2 was able to change the organization of hyphae, reducing hyphae branches and ECM production (Figure 5D).

### 3.5 *Ps*d2 Causes Morphological Changes in A. nidulans Biofilms

**FIGURE 6:**
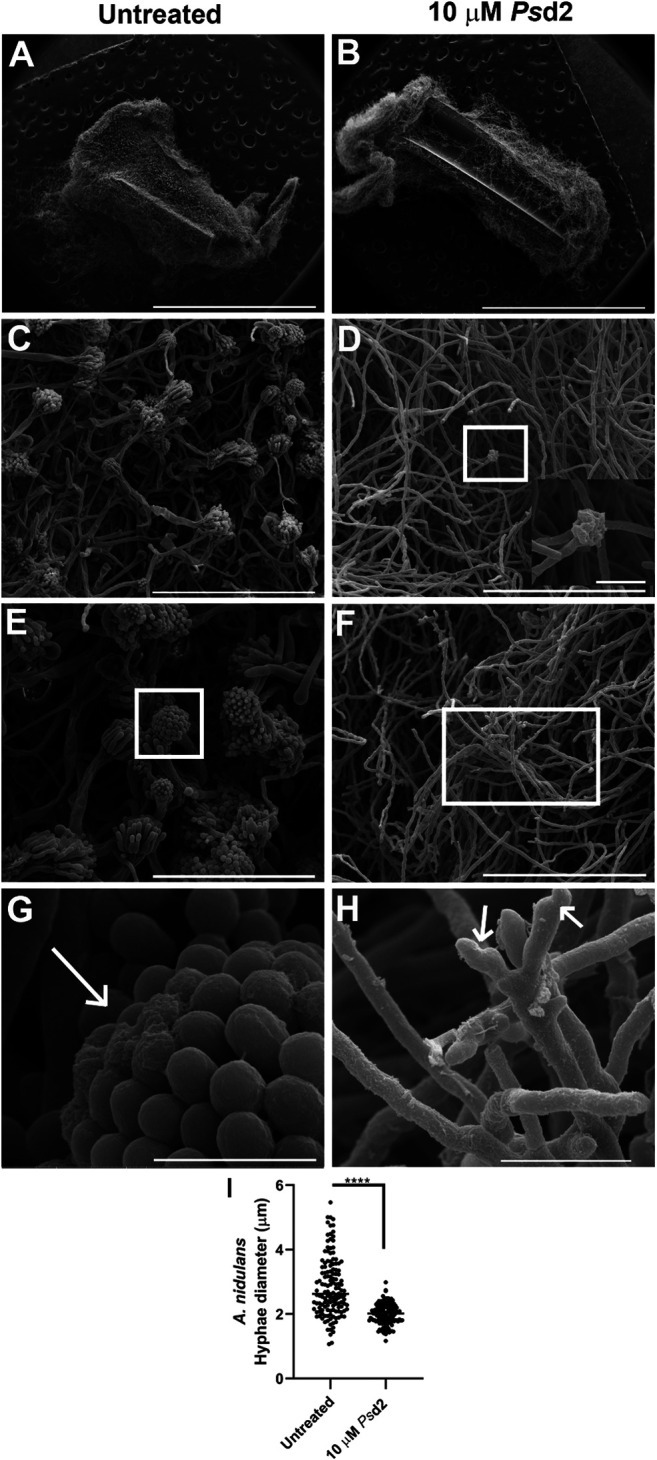
Scanning electron micrographs of ultrastructural changes in *A. nidulans* biofilms induced by *Ps*d2 defensin. *A. nidulans* biofilms were grown in YUU medium for 24 h at 37°C in CVCs in the absence **(A,C,E,G)** or presence of 10 µM *Ps*d2 **(B,D,F,H)**. The inset zoom image is at 12000 × magnification **(D)** and represents a deformed conidiophore in the biofilm treated with 10 µM *Ps*d2. The white arrows represent ECM accumulated in the conidiophore **(G)** and deformed hyphae **(H)**. Scale bars represent 3 mm **(A,B)**, 100 µm **(C,D,F)**, 50 µm **(E)**, 10 µm **((H)**, inset zoom image), 5 µm **(G)**. Hyphae diameter **(I)** of the untreated and treated biofilms was measured using ImageJ software. Significant differences (*p* < 0.05) are represented by asterisks; ****p < 0.0001.

SEM microscopic analysis was performed to investigate the ultrastructural changes induced by *Ps*d2 defensin in *A. nidulans* biofilm. *A. nidulans* biofilm was grown in the absence or presence of 10 µM *Ps*d2 in sections of 0.5 cm of CVCs placed in polystyrene 96-well plates. SEM micrographs showed a multicellular biofilm with a dense network (Figures 6A,C). A significant number of mature conidiophores (Figure 6C) and the presence of covered ECM (long arrow in Figure 6G) were also observed in untreated biofilms. Treatment with 10 µM *Ps*d2 for 24 h led to substantial damage and reduction of conidiophores (Figure 6D) and *A. nidulans* hyphae (short arrows in Figure 6H). Furthermore, a significant difference was detected in the hyphae diameter of *Ps*d2-treated biofilms compared to untreated ones (*p* < 0.0001) (Figure 6I).

## 4 Discussion

The foundation for this study was based on two questions. The first was whether *A. nidulans* was able to adhere, grow, and develop an ECM-producer biofilm, which is one of the most important virulence factors of *C. albicans* and *A. fumigatus* (González-Ramírez et al., 2016; Khan et al., 2020; Mohammadi et al., 2020). The second was whether the pea defensin *Ps*d2 would inhibit and cause structural changes to *A. nidulans* biofilms.

The first approach was to establish the best medium for *A. nidulans* biofilm growth and to analyze the early stages of its development. Optical microscopy assays were performed to observe the biofilm adhesion, germination, branching, and development. We found that *A. nidulans* grown in RPMI UU and YUU media adhered to the polystyrene surface at 4 h, compared to those grown in the RPMI medium. Subsequently, *A. nidulans* germinated and colonized the polystyrene surface at 8 h (Figure 1B,C). The attachment of *A. nidulans* conidia on polystyrene was vital for biofilm development in RPMI UU and YUU media and was a prerequisite for the initiation of biofilm development, as previously observed in *C. albicans, A. niger,* and *A. fumigatus* biofilms (Wächtler et al., 2011; de Groot et al., 2013; Croft et al., 2016; Chatterjee and Das, 2019; Martin et al., 2021). Previous studies using CLSM and SEM showed that *A. fumigatus* grown at 28°C and 37°C (González-Ramírez et al., 2016) and *A. niger* grown at 25°C (Chatterjee and Das, 2019), adhered at 4 h to the polystyrene and glass surfaces, respectively. Interestingly, in both *A. niger* and *A. fumigatus* conidia, we observe the presence of extracellular polymeric substances (EPS) covering the conidia surface (González-Ramírez et al., 2016; Chatterjee and Das, 2019). During the attachment stage, physical contact and active attachment between the conidia and the surface and the conidium-conidium was executed (González-Ramírez et al., 2016). The initial adhesion may be attributed to the presence of hydrophobins in the spore surface, which are critical ubiquitous proteins for the initial colonization of filamentous fungi via adhesion to abiotic and biotic surfaces, as previously shown for *A. fumigatus, A. niger, and A. nidulans* as well as other *Aspergillus* species (Dague et al., 2008; Jensen et al., 2010; de Groot et al., 2013)*.* This hydrophobic characteristic governs primary host-pathogen interactions, and they could be involved in the adhesion on the surface of the polystyrene plate and the CVCs, initiating biofilm formation (de Groot et al., 2013; Grünbacher et al., 2014). To quantify the biofilm formation over time, we used complementary assays, including XTT-reduction, crystal violet, and optical density. XTT-reduction assay is often used to quantify *C. albicans* (Silva et al., 2008; Lohse et al., 2017; Curvelo et al., 2019), *A. fumigatus* (Seidler et al., 2008; Mowat et al., 2009)*, Scedosporium spp.* (Rollin-Pinheiro et al., 2017; Mello et al., 2018; Rollin-Pinheiro et al., 2019) and *Cryptococcus spp.* (Martinez et al., 2008; Martinez et al., 2011) biofilms growth *in vitro* and to evaluate the viability of biofilms after treatment with antimicrobial agents, which can be correlated with levels of cell density and metabolic activity (Mowat et al., 2009; Lohse et al., 2017). However, if alterations in the metabolic profile during the phases of the biofilm development occur, the use of XTT to assess mature biofilms can lead to inaccuracies because cell layers at the bottom of the biofilm become quiescent and inaccessible to the XTT (Pierce et al., 2017). We found that *A. nidulans’* metabolic activity and biomass production had similar profiles for all tested media (RPMI, RPMI UU, and YUU) (Figures 2B,C). In contrast, higher *A. nidulans* absorbance levels in YUU were achieved than those grown in the RPMI UU medium in optical density analysis (Figure 2A). One possible explanation for this discrepancy is that optical density is a direct and physical biofilm measurement that is a strict measure that avoids several processing steps that can cause cell loss as biofilms peel off from the bottom of wells due to innumerous washing steps (Pierce et al., 2008; Lohse et al., 2017). For example, Lohse et al. suggested that using many assays simultaneously for quantification would provide the most comprehensive analysis for *C. albicans* biofilms, supporting the notion that cell density analysis is a high-throughput methodology and must be combined with other techniques (Lohse et al., 2017). Thus, because the absorbance levels measurement by Cytation-5 (Biotek) was an uninterrupted process, there was no data loss through the biofilm formation. Indeed, other quantification assays such as XTT-reduction and crystal violet staining used in this study were incapable of accessing the increased biomass of *A. nidulans* biofilm in the YUU medium, which required the establishment of another method to quantify the biofilm growth. Hence, our data converge with previous findings and discussions (Pierce et al., 2008; Lohse et al., 2017). Here, we support the idea of meticulously using two or more techniques to quantify biofilm development over time.

To analyze the structural organization in early and late periods of the *A. nidulans* biofilms formation, SDCM was performed. The biofilms were grown until 24, 39, and 48 h and stained with Calcofluor white and Filmtracer Sypro Ruby and then visualized using SDCM. The presence of a dense and thick biofilm that became more complex and coarse over time was revealed (24, 39, and 48 h, Figure 3), corresponding to the exponential phase of growth as previously observed in the biofilm quantification graphs (Figures 2A,B,C). As Figures 3A,B,C illustrated, the biofilm mainly increased the depth of the network that could be explained by the vertically polarized growth and the presence of water channels among the groups of hyphae. These characteristics have been reported for *A. niger* biofilms grown on polyester cloth and stained with fluorescein isothiocyanate (FITC), which binds to proteins (Villena et al., 2010) and *A. fumigatus* biofilms grown in polystyrene plates (Beauvais et al., 2007; González-Ramírez et al., 2016)*,* visualized using the SDCM and SEM techniques, respectively. The staining also detected the production of ECM with Filmtracer Sypro Ruby, the primary feature described for the biofilm formation in *C. albicans* (Fanning and Mitchell, 2012; Pierce et al., 2017), and *A. fumigatus* (Beauvais et al., 2007; Harding et al., 2009; Beauvais and Latgé, 2015). The ECM is responsible for maintaining adhesive and cohesive interactions, providing mechanical stability to biofilms, controlling even the detachment of cells from the biofilm, which is crucial to dispersal and colonization on other surfaces and restarting the cycle of the biofilm formation, as previously found in *A. fumigatus* and *C. albicans* biofilms (Harding et al., 2009; Pierce et al., 2017). The most clinically relevant function associated with the biofilm matrix is its role in antifungal resistance in pathogenic fungi such as *C. albicans* and *A. fumigatus* (Mitchell et al., 2016b; Reichhardt et al., 2016). In *C. albicans*, fluconazole was sequestered by ECM, which was correlated with the quantity of β-1,3-glucans in the matrix (Nett et al., 2007). As previously found by Beauvais et al. (2007) and Seidler et al. (2008) in *A. fumigatus* biofilms and by Villena et al. (2010) and Chatterjee and Das (2019) in *A. niger* biofilms, the dry weight and the ECM increased from 24 to 48 h of growth. These findings corroborate our present findings, in which an increase of the total biomass was followed by the formation of a thicker ECM (Beauvais et al., 2007; Seidler et al., 2008). Over time, conventional antibiotic therapy has become less effective due to developing mechanisms for resistance among fungal species (Osherov et al., 2001; Lockhart et al., 2011; Fanning and Mitchell, 2012; Lockhart and Guarner, 2019). In this context, plant defensins might be a promising tool in developing novel antimycotics. Previous works have already described the effects of defensins on fungal growth (Almeida et al., 2000; Aerts et al., 2007; Weerden et al., 2010; Cools et al., 2017). *Ps*d2 is a pea defensin that interacts and depends on the glucosylceramide and ergosterol organized in membrane rafts of *A. nidulans* to exert its biological activity. In addition, the defensin is non-toxic to erythrocytes (Fernandes et al., 2016; Amaral et al., 2019).

To answer our second question, we tested the action of plant defensin *Ps*d2 against *A. nidulans* biofilms. *Ps*d2 reduced biofilm colonization and growth over time in a dose-dependent manner (Figure 4A). XTT-reduction, crystal violet, and safranin O quantifications (Figures 4B,C,D) indicated that *Ps*d2 reduced *A. nidulans* cell viability and biomass production by 50% and ECM by 40%. The ability of *Ps*d2 to inhibit biofilm development and promote structural changes was analyzed using SDCM and SEM (Figures 5, 6). *Ps*d2 at 10 μM led to structural modifications such as inhibiting conidiophores formation and impairing conidia formation, reducing hyphae length and diameter. To the best of our knowledge, previous studies focused only on testing plant defensins against *C. albicans* biofilms. (Vriens et al., 2015), the pioneer in those studies found that *Hs*AFP1, a defensin isolated from coral bells (*Heuchera sanguinea*), inhibited *C. albicans* biofilm development by 50% at 11 μM by preventing hyphae network. Likewise (Vriens et al., 2016), demonstrated that *Rs*AFP2 isolated from *Raphanus sativus* inhibited *C. albicans* biofilms at 288 μM. Similar to what is found for *Ps*d2, *Rs*AFP2 depends on the interaction with glucosylceramide from the fungal membrane to exercise its inhibitory activity that leads to fungal death (Thevissen et al., 2004; Thevissen et al., 2012). Because *Rs*AFP2 impaired yeast-to-hypha transition in *C. albicans*, the authors suggested that, after the interaction of *Rs*AFP2 with the glucosylceramide, the defensin induced a weakening of the biofilm cells or biofilm matrix that could not progress to a mature biofilm (Vriens et al., 2016). Like plant defensins, monoclonal antibodies and inhibitors that bind to glucosylceramide interfered with cell replication and reduced adhesion and germination to polystyrene surfaces in *C. albicans, Fonsecaea spp.*, and *Scedosporium spp*. (Nimrichter et al., 2004; Rollin-Pinheiro et al., 2019; Rochetti et al., 2020; Rollin-Pinheiro et al., 2021). These lines of evidence highlight the critical role of sphingolipids and lipid rafts as targets for inhibiting pathogenic fungi growth and biofilm formation.

Our findings offer a new perspective on the ways plant defensins inhibit filamentous fungal biofilms. We hypothesized that *Ps*d2 interacted with membrane rafts enriched with glucosylceramide and ergosterol of *A. nidulans* biofilm and inhibited growth by preventing the development of conidiophores, which is required for reproduction and consequent maintenance of biofilm growth. One possible explanation is that, after the interaction of *Ps*d2 with the glucosylceramide and ergosterol and subsequent accumulation, the plasma membrane could be weakened and disrupted, which could be explained by the volume loss observed in treated cells (Figure 6). This effect was also achieved in *C. albicans* biofilms treated with 20 μM *Ps*d1 (Gonçalves et al., 2017), another pea defensin isolated and characterized by our group that shares 44.7% of identity in the primary sequence with *Ps*d2 defensin (Almeida et al., 2000). By visualizing the biofilm by atomic force microscopy, the authors suggested that *Ps*d1 first acted on the fungal wall, disaggregating the polysaccharide matrix and disturbing the wall. It affected the integrity of the cell wall by increasing cell roughness and decreasing its rigidity. When the peptide reached the cell membrane, it interacted with glucosylceramides in the *C. albicans* membrane and induced an intracellular effect. Subsequently, the intracellular accumulation of *Ps*d1 interfered with the cell cycle control protein cyclin F, as previously described for *C. albicans* planktonic cells (De Medeiros et al., 2014), in a similar way found for *N. crassa* (Lobo et al., 2007), which led to apoptosis of the fungal pathogen (Gonçalves et al., 2017). *Ps*d1 caused a lower decrease in the rigidity of ∆gcs *C. albicans* cell surface than the wild-type strain, which could be explained by the strong evidence that *Ps*d1 had glucosylceramide as a molecular target in *C. albicans* membrane, as previously suggested in planktonic cells (De Medeiros et al., 2010; De Medeiros et al., 2014; Gonçalves et al., 2017). Nevertheless, further studies are necessary to determine whether *Ps*d2 disturbs the plasma membrane integrity of *A. nidulans* and reaches intracellular targets as well as *Ps*d1 defensin.

Currently, three classes of antifungal compounds are commonly used to treat *Aspergillus spp.* infections: triazoles, polyenes, and echinocandins (Chang et al., 2017; Perlin, 2017; Shishodia et al., 2019). These agents target the cell wall either by disrupting ergosterol or β-1,3-glucan biosynthesis or by targeting the ergosterol molecule directly, which are essential components of the fungal membrane (Chang et al., 2017). Nevertheless, azole-resistant isolates *Aspergillus spp*. have been highly identified in the last decade (Vermeulen et al., 2013; Fisher et al., 2018; Shishodia et al., 2019). In general, these resistant isolates are reported when the minimal inhibitory concentration (MIC) values are above the epidemiologic cutoff values based on the European Committee on Antimicrobial Susceptibility Testing (EUCAST) or the Clinical and Laboratory Standards Institute (CLSI) defined breakpoints for *Aspergillus spp.* (Shishodia et al., 2019)*.* For instance, Espinel-Ingrof et al. (2010) reported an increase of the MIC for voriconazole in different wild-type strains of *Aspergillus spp*., as *A. fumigatus* that reached the concentration at 22 μM, which exceeded EUCAST (MIC ≤2.7 µM) and CLSI (MIC ≤5.7 µM) cutoff values for voriconazole defined in 2020 (CLSI, 2020; mEUCAST, 2020; Espinel-Ingroff et al., 2010; Shishodia et al., 2019; Pfaller et al., 2021). This evidence of triazole resistance was also extended to other species, especially in *A. niger*, *A. versicolor,* and *A. nidulans*. Thus, antifungal resistance can lead to long-term and less effective treatments, as well as more intense side effects such as visual disturbances, hepatotoxicity, skin rashes, and neurotoxicity, which eventually lead to discontinuation of the treatment (Racette et al., 2005; Zonios et al., 2014; Zhong et al., 2018).

Even though 10 μM *Ps*d2 defensin inhibited 50% of the cell viability and global biomass and 40% of ECM of the *A. nidulans* biofilm, we suggest its potential use as a prototype to design new antifungal agents, mainly due to its different mechanism of action compared to the other antifungal agents used currently (Perlin, 2017; Amaral et al., 2019). Previous works of our group showed the specific interaction of *Ps*d1 and *Ps*d2 with membrane rafts enriched with glucosylceramide and ergosterol and also the internalization of *Ps*d1 (Lobo et al., 2007; De Medeiros et al., 2014; Fernandes et al., 2016; Amaral et al., 2019; Do Amaral et al., 2020). In this respect, some strategies could be followed, such as synthesizing smaller analogs derived from *Ps*d2 with an improved therapeutic index and combining therapy (Tits et al., 2020). Combining antimicrobial peptides and drugs that possess different targets is based on the potentiation of the result in a widened drug activity spectrum, lower doses of toxic drugs, quicker antifungal action, and a lower risk for the occurrence of fungal drug resistance (Tits et al., 2020). Therefore, combination therapy with a synergic response can offer an excellent alternative approach (Shishodia et al., 2019; Tits et al., 2020). Antibiofilm activity combining *Ps*d2 defensin and azole compounds, such as itraconazole and voriconazole in *A. nidulans* biofilms in YUU medium, has been performed by our group, but additional studies/assays are necessary to evaluate their synergic potential.

In summary, we demonstrated that *A. nidulans* adheres to abiotic surfaces and grows as a complex, robust, multicellular biofilm, producing an ECM as it matures. *Ps*d2 defensin inhibits this biofilm, reducing the ECM levels and leading to critical changes in biofilm maintenance. Because *Ps*d2 defensin has selective activity against fungi, interacting with membrane raft domains enriched in glucosylceramide and ergosterol and does not show hemolytic activity for mammalian cells, our findings offer a new perspective of *Ps*d2; it is a promising candidate for the treatment of fungal infections caused by *A. nidulans*. Future research may extend this work by understanding the *Ps*d2 mechanisms of action on *A. nidulans* biofilms.

## Data Availability

The raw data supporting the conclusion of this article will be made available by the authors, without undue reservation.
